# Active Packaging Films from PBAT/PLA with *Rosmarinus officinalis* L. Extract: Antioxidant, UV-Shielding, and Compostable Properties

**DOI:** 10.3390/molecules31020217

**Published:** 2026-01-08

**Authors:** Xiaoyan He, Lisheng Tang, Ran Huang

**Affiliations:** 1 Provincial Intelligent Health Proof-of-Concept Center, Yiwu Research Institute of Fudan University, Yiwu 322000, China; xiaoyanhe@rizt.ac.cn (X.H.); tangls@rizt.ac.cn (L.T.); 2 Zhuhai Fudan Innovation Research Institute, Zhuhai 519031, China; 3 Yiwu Research Institute, Fudan University, Shanghai 200433, China; 4 Center for Innovation and Entrepreneurship, Taizhou Institute of Zhejiang University, Taizhou 318000, China

**Keywords:** PBAT/PLA film, rosemary extract, biodegradable packaging, antioxidant, UV-blocking

## Abstract

With the growing demand for eco-friendly food packaging, poly(butylene adipate-co-terephthalate) (PBAT)/polylactic acid (PLA) composite films have emerged as promising biodegradable alternatives, but their inherent limitations (e.g., poor antioxidant capacity, weak UV stability, and insufficient antimicrobial activity) hinder practical applications. This study aimed to address these drawbacks by incorporating *Rosmarinus officinalis* L. extract (RM) as a natural multifunctional additive. PBAT/PLA/RM blend films with RM concentrations of 0.1%, 0.3%, 0.5%, and 1% were fabricated via melt extrusion and blown film processing. Key characterizations were conducted to evaluate thermal stability, mechanical properties, morphology, antioxidant activity, UV-shielding performance, antimicrobial efficacy, and biodegradability. The results showed that RM significantly enhanced the antioxidant capacity of the films, with the highest DPPH radical scavenging activity achieved at 0.3% RM. UV-blocking performance improved incrementally with increasing RM concentration, and films containing ≥0.5% RM filtered over 90% of UVA and UVB radiation. All composite films met biodegradability standards, with over 90% degradation observed after 240 days of composting, though RM prolonged the initial degradation stage by inhibiting early microbial activity. However, the antimicrobial effect of RM was limited, and concentrations exceeding 1% caused film stickiness, impeding processing. This work demonstrates that RM is a viable natural additive for functionalizing PBAT/PLA films, offering enhanced antioxidant and UV-shielding properties while maintaining biodegradability, thus providing a promising solution for sustainable food packaging.

## 1. Introduction

In recent years, the environmental impact of synthetic plastics has led to a surge in the development of biodegradable alternatives [1]. Among these, polylactic acid (PLA) and poly(butylene adipate-co-terephthalate) (PBAT) have emerged as promising candidates due to their biodegradability and potential for reducing plastic waste [2]. PLA is a thermoplastic aliphatic polyester derived from renewable resources such as corn starch or sugarcane, making it an eco-friendly alternative to petroleum-based plastics. It is known for its high strength, rigidity, and clarity, which makes it suitable for various applications, including food packaging [3]. PBAT, on the other hand, is a synthetic copolymer that combines the benefits of both aliphatic and aromatic polyesters. It is known for its flexibility, toughness, and biodegradability, which complement the brittleness and rigidity of PLA [4]. The combination of PLA and PBAT results in a packaging system that leverages the strengths of both materials: the eco-friendliness and strength of PLA, and the flexibility and durability of PBAT. This synergy has led to increased research and industrial interest in PLA/PBAT composite films for food packaging applications [5].

However, despite the advantageous properties of PLA/PBAT composites, there are still some limitations that need to be addressed [6,7]. One of the main challenges is the films’ limited antimicrobial activity, which is crucial for ensuring the safety and extending the shelf life of packaged foods [8]. PLA/PBAT films also face challenges related to UV stability and antioxidant properties. These limitations can lead to faster degradation of the film when exposed to light and a reduced ability to prevent oxidation of the packaged food, respectively [9].

To overcome these challenges, researchers have turned their attention to natural additives that can enhance the properties of PLA/PBAT films [10]. Natural additives are attractive because they are typically biocompatible, non-toxic, and derived from renewable resources, while a number of artificial chemical additives are not suitable to be added in food-contact products. Among these, plant extracts have gained significant interest due to their rich content of bioactive compounds, which can impart additional functionalities such as antimicrobial, antioxidant, and UV-blocking properties [9].

Many natural extracts, such as essential oils, tannins, and polyphenols, have been explored for their potential to improve the performance of biodegradable films. These natural compounds can interact with the polymer matrix, enhancing its mechanical, thermal, and barrier properties. However, finding the right balance between improving these properties and maintaining the biodegradability and processability of the films remains a challenge [11,12]. Therefore, the selection of an appropriate natural additive is crucial for developing a high-performance PLA/PBAT composite film [13].

Rosemary has a long history of cultivation and consumption. Studies have confirmed that rosemary is rich in a variety of polyphenols, such as rhamnoside, rhamnol, rosmarinol, and rosmarinic acid, and has a variety of physiological active functions, such as antioxidant, antibacterial, anti-inflammatory, anti-tumor, and anti-gastric ulcer [14]. As a natural, safe and efficient antioxidant and antimicrobial agent, rosemary extract (Rosmarinus officinalis) is currently approved as a food additive in many countries and regions, and its application in the field of food preservation and safety control has received widespread attention [15,16].

Furthermore, rosemary extract has emerged as a promising natural additive for enhancing the properties of biodegradable films. The above-mentioned bioactive compounds contained can provide multiple benefits when incorporated into PLA/PBAT films. Additionally, rosemary extract has been shown to possess UV-blocking properties. The phenolic compounds in the extract can absorb UV radiation, thereby protecting the packaged food from UV-induced degradation. This additional functionality can be particularly beneficial for packaging applications where the product is exposed to light [17].

Given the promising properties of rosemary extract, this study aims to develop and evaluate PLA/PBAT composite films incorporating rosemary extract as a natural additive. The motivation behind this work is to create a biodegradable packaging film that not only maintains the desirable properties of PLA and PBAT but also addresses their limitations by leveraging the antioxidant, antimicrobial, and UV-blocking capabilities of rosemary extract. In this work, PBAT and PLA were used as packaging film substrates, and PBAT/PLA/rosemary extract composite films were prepared by melt extrusion pelletizing and blown film processing, and the various properties of the films were investigated separately. We have: (1) investigated the effect of rosemary extract on the thermal stability, mechanical properties, and barrier properties of PLA/PBAT films; (2) evaluated the antioxidant and antimicrobial efficacy of the composite films to determine their potential for enhancing food safety and shelf life; (3) assessed the UV-blocking properties and biodegradability of the films to ensure they meet the requirements for sustainable and effective food packaging. By achieving these objectives, this work aims to contribute to the development of high-performance, eco-friendly food packaging solutions that can help reduce the environmental impact of plastic waste while ensuring the safety and quality of packaged foods.

Although natural plant extracts have been previously incorporated into biodegradable polymer films, most existing studies rely on solvent-casting methods and focus on isolated functional properties. In contrast, the present work investigates the incorporation of rosemary extract into PBAT/PLA films using industrially relevant melt extrusion and blown-film processing, which better reflects practical food-packaging manufacturing conditions. Moreover, this study provides a systematic and integrated evaluation of the effects of rosemary extract on film structure, thermal behavior, optical properties, antioxidant and antibacterial performance, and time-resolved compost biodegradation. Notably, the influence of rosemary extract on the early-stage biodegradation kinetics of PBAT/PLA films is examined in detail, revealing a transient inhibition of microbial activity followed by full compostability. These results offer new insights into the multifunctional role of rosemary extract in PBAT/PLA packaging systems and contribute to the rational design of active biodegradable films for food-contact applications.

## 2. Results and Discussion

### 2.1. Components Analysis of Rosemary Extracts

#### 2.1.1. FTIR

**Figure 1:**
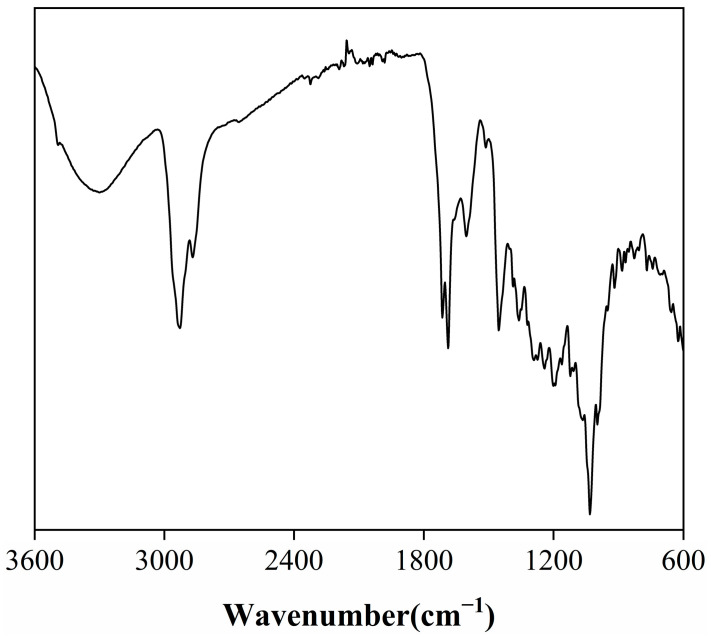
FTIR spectra of rosemary extract.

The Fourier-transform infrared (FTIR) spectrum of the rosemary extract (Figure 1) reveals a series of characteristic absorption bands that reflect its rich functional diversity and underlying chemical composition. A broad peak around 3300 cm^−1^ corresponds to O–H stretching vibrations of hydroxyl groups, confirming the presence of phenolic and alcoholic compounds, which are primarily responsible for the extract’s antioxidant and radical-scavenging activity [18]. Additional peaks near 1120 cm^−1^ and 1107 cm^−1^, attributed to C–O stretching and O–H in-plane bending, together with a band around 1360 cm^−1^, further indicate the abundance of hydroxyl functionalities associated with polyphenols. The absorption band at 2928 cm^−1^ arises from C–H stretching vibrations of aliphatic –CH_2_ and –CH_3_ groups, characteristic of alkyl chains within terpenoids and other hydrocarbons. Two notable carbonyl absorptions at 1714 cm^−1^ and 1687 cm^−1^ correspond to C=O stretching of ketones, aldehydes, and carboxylic acids, suggesting a substantial presence of oxygenated compounds [19]. Meanwhile, a series of bands at 1600 cm^−1^, 1513 cm^−1^, 1454 cm^−1^, and 1386 cm^−1^ can be assigned to aromatic C=C stretching vibrations, indicative of conjugated aromatic systems such as phenolic diterpenoids and flavonoids [18,20]. The smaller band near 1273 cm^−1^ further implies aromatic ester linkages, likely originating from esterified phenolic compounds. The FTIR findings confirm that the extract consists predominantly of hydroxyl-, carbonyl-, and aromatic-bearing molecules, consistent with bioactive terpenoids and phenolic derivatives known for their thermal and oxidative stability [21].

#### 2.1.2. TGA

**Figure 2:**
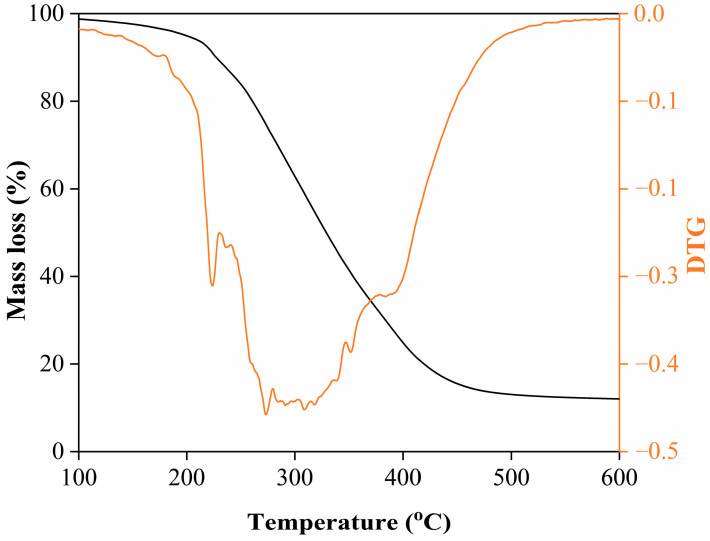
TGA and DTG (colored line) curves of rosemary extract.

The thermal stability and decomposition behavior of the rosemary extract were evaluated using thermogravimetric analysis (TGA), as presented in Figure 2. The TGA curve displays a clear three-stage weight-loss pattern, reflecting the stepwise decomposition of different chemical fractions within the extract.

The first stage, between 25 and 150 °C, corresponds to a minor mass loss of approximately 5–8%, primarily attributed to the evaporation of physically adsorbed water and low-molecular-weight volatile compounds such as light terpenes and small aromatic molecules. This limited loss at low temperature demonstrates that the extract remains chemically stable during moderate heating, suggesting that it can be safely applied in low-temperature processes such as blending with edible oils or incorporation into cosmetic formulations without significant volatilization or degradation of its bioactive components [22,23].

The second stage, spanning 200–400 °C, represents the main decomposition phase, accounting for 60–70% of the total weight loss. This substantial decline is associated with the thermal degradation of the principal organic constituents—notably phenolic compounds, terpenoids, and carbonyl-bearing molecules identified by FTIR. These compounds undergo oxidative breakdown, decarboxylation, and polymeric rearrangements at elevated temperatures, leading to a steep mass reduction. The onset of significant degradation above 200 °C suggests that thermal processing beyond this range would cause irreversible loss of key antioxidant compounds, thereby diminishing the extract’s functional properties [22,24].

At temperatures above 400 °C, the TGA curve levels off, leaving a 10–15% residual mass at 600 °C, which corresponds to the formation of thermally stable char residues. These residues mainly consist of carbonized aromatic structures derived from polyphenolic and flavonoid frameworks. The persistence of this char fraction indicates the high aromatic stability of the extract, as these condensed structures resist complete decomposition even at elevated temperatures under inert conditions [25]. Overall, the TGA results confirm that the rosemary extract possesses good thermal stability below 200 °C, making it suitable for medium-temperature applications while highlighting its sensitivity to prolonged high-temperature exposure.

#### 2.1.3. Chemical Analysis

The chemical composition of the rosemary extract was further elucidated by gas chromatography–mass spectrometry (GC–MS). A total of 15 compounds were identified based on spectral matching with the NIST database (Table 1), encompassing volatile hydrocarbons, terpenoids, phenolic compounds, flavonoids, and vitamin derivatives, each contributing distinctively to the extract’s physicochemical and bioactive properties.

The early-eluting volatile components, including 3-pentanone (3.59%), 1-ethoxybutane (4.14%), and octane (24.59%), correspond to the low-temperature weight loss observed in the TGA curve and the aliphatic C–H stretching band at 2928 cm^−1^ in the FTIR spectrum. These volatile constituents contribute primarily to the extract’s aroma and reflect its fraction of light, low-polarity molecules [26,27].

The extract also contains a rich array of terpenoids, which represent its core bioactive components. Detected compounds include (1S-endo)-1,7,7-trimethylbicyclo[2.2.1]heptan-2-ol (0.67%), 4,6,6-trimethylbicyclo[3.1.1]hept-3-en-2-one (3.08%), 14-hydroxycaryophyllene (0.54%), isocarnosol (22.59%), and 12-O-methylcarnosol (1.44%). Among them, isocarnosol exhibits the largest peak area, confirming its dominance as a major diterpenoid antioxidant. These terpenoid structures correspond well with the hydroxyl and carbonyl peaks observed in FTIR and the main decomposition range of the TGA profile, indicating their contribution to the extract’s bioactivity and thermal behavior [28,29,30].

In addition to terpenoids, several phenolic compounds were detected, such as ferruginol (2.05%), 2,6-bis(1,1-dimethylethyl)-4-[(4-hydroxy-3,5-dimethylphenyl)methyl]phenol (7.72%), and the trimethylsilyl (TMS) derivative of 4-cumylphenol (21.65%). The derivatization of phenolic hydroxyl groups enhances volatility, allowing for their GC–MS detection. The high abundance of these alkyl-substituted phenols is consistent with the strong aromatic and hydroxyl peaks identified by FTIR [28]. Furthermore, flavonoids such as 5-hydroxy-7-methoxy-2-(4-methoxyphenyl)-4H-1-benzopyran-4-one (3.19%) and 5-hydroxy-6,7-dimethoxy-2-(4-methoxyphenyl)-4H-1-benzopyran-4-one (1.81%), along with dl-α-tocopherol (1.53%), were detected at higher retention times due to their larger molecular weights and lower volatility. Although present in smaller quantities, these compounds contribute synergistically to the overall antioxidant capacity of the extract through hydrogen donation and radical stabilization mechanisms [27,30,31].

Overall, the GC–MS results corroborate the FTIR and TGA findings, confirming that the rosemary extract predominantly contains phenolic diterpenoids, terpenoids, and alkyl-substituted phenols, which not only account for its strong antioxidant behavior but also explain its characteristic thermal decomposition pattern.

### 2.2. Thermal Stability

**Figure 3:**
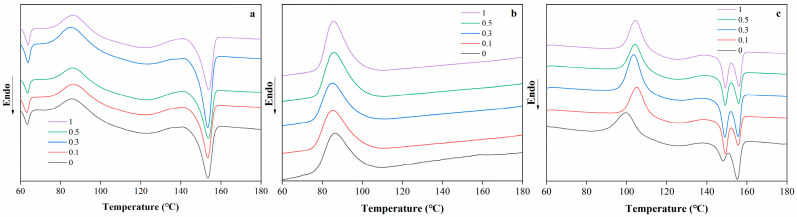
DSC thermograms of PBAT/PLA/RM films, heating scan (**a**), cooling scan (**b**), reheating scan (**c**).

The DSC thermograms of PBAT/PLA blend films with different RM concentrations are shown in Figure 3. The blend films exhibited distinct thermal behavior as a function of temperature (ranging from 60 to 180 °C). For the neat PBAT/PLA films, characteristic thermal transitions corresponding to the melting or glass transition of PLA and PBAT are observed. With the incorporation of RM, noticeable shifts in the thermal transition temperatures and changes in the enthalpy-related peaks were detected. At lower RM concentrations (0.1% and 0.3%), the thermal transition peaks remain relatively close to those of the neat film, indicating minimal disruption to the crystalline structure of the polymers. However, as the RM concentration increases to 0.5% and 1%, slight broadening or shifting of the peaks was observed, which may be attributed to the interfacial interactions between RM and the PBAT/PLA matrix.

The presence of RM was hypothesized to influence the crystallization kinetics of the blend: RM may act as a nucleating agent or interfere with chain mobility during crystallization. Similar to the mechanism reported in compatibilized polymer blends [32], the epoxy groups in RM could react with the carboxyl or hydroxyl groups of PLA and PBAT, leading to the formation of graft copolymers at the interface. This interfacial modification might alter the segmental motion of the polymer chains, thereby affecting their thermal transitions. The subtle changes in the DSC curves suggest that RM concentration plays a regulatory role in the thermal behavior of PBAT/PLA blends, with higher concentrations inducing more pronounced interfacial effects on the crystalline structure.

**Figure 4:**
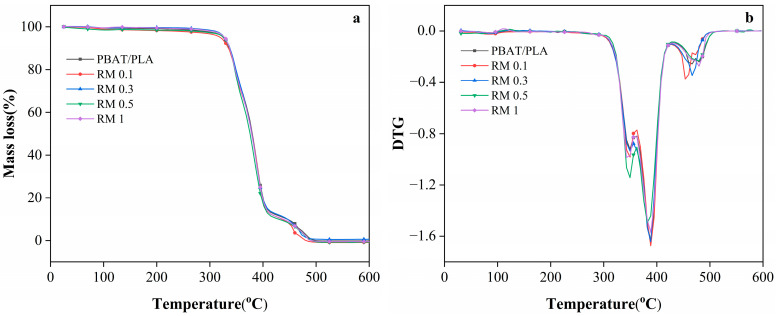
The TGA (**a**) and DTG (**b**) behavior of PBAT/PLA/RM films during the thermal decomposition.

The TGA curves (Figure 4) and Table 2 (characteristic decomposition temperature) provide insights into the thermal stability of PBAT/PLA blends modified with RM. The onset decomposition temperature (T_onset_) of the neat PBAT/PLA blend is 323.5 °C, which is consistent with the thermal stability of typical PLA/PBAT blends reported in the literature. When 0.1% RM was added, T_onset_ decreased slightly to 317 °C, possibly due to the initial disruption of the polymer matrix by low RM content, which may accelerate the early-stage decomposition. However, as the RM concentration increased to 0.3%, 0.5%, and 1%, T_onset_ reverts to 323.5 °C, indicating improved thermal stability at higher RM loadings. This recovery of thermal stability was likely associated with the chain extension or branching reactions induced by RM: the epoxy-functionalized RM can form cross-links or longer polymer chains with PLA and PBAT, enhancing the resistance to thermal degradation [33,34].

The multiple melting/decomposition temperatures (T_m1_–T_m4_) in Table 2 reflected the stepwise decomposition of the blend components. For the neat PBAT/PLA films, T_m3_ and T_m4_ (466.5 °C and 479.5 °C) corresponded to the decomposition of the more thermally stable segments of PLA and PBAT. With 0.1% RM, T_m3_ decreased to 453.5 °C, while T_m4_ dropped to 473 °C, suggesting weakened thermal stability of these segments. In contrast, blends with 0.3% RM retained T_m3_ at 466.5 °C, similar to the neat blend, indicating that sufficient RM content can mitigate the decomposition of the polymer segments. For films with 0.5% and 1% RM, T_m4_ remained at 479.5 °C, further confirming that higher RM concentrations promote the formation of a more thermally stable network structure via interfacial reactions. The disappearance of T_m4_ in blends with RM concentrations ≥0.3% may be due to the formation of a homogeneous interfacial region, where the distinct decomposition of individual polymer segments is suppressed by the graft copolymers (PLA-RM-PBAT) formed in situ.

### 2.3. Mechanical Property

**Figure 5:**
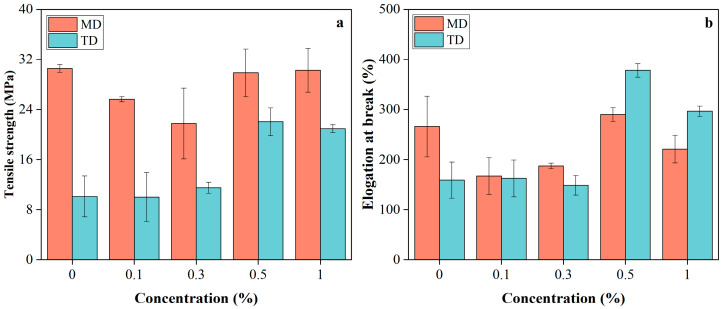
Tensile strength (**a**) and elongation at break (**b**) of PBAT/PLA/RM films.

The mechanical properties of PBAT/PLA/RM films (Figure 5) showed dependence on RM concentration and testing direction (MD: machine direction; TD: transverse direction), reflecting the combined effects of blown-film-induced anisotropy and RM dispersion within the matrix. In the machine direction (MD), increasing RM content from 0 to 0.3% led to reduced tensile strength and elongation at break, likely due to poor RM dispersion and microaggregation, which introduced stress concentration sites that disrupted stress transfer along the oriented polymer chains. With further RM addition to 0.5–1%, these properties recovered to levels comparable to the pristine film, suggesting improved RM dispersion and enhanced interfacial interactions between RM and the PBAT/PLA matrix; weak interactions such as hydrogen bonding between RM polar groups and polymer ester groups likely contributed to restoring stress-transfer continuity. In contrast, in the transverse direction (TD), tensile properties remained nearly unchanged at low RM contents (0–0.3%), indicating that the randomly entangled chain structure is less sensitive to localized defects, whereas at higher RM loadings (0.5–1%), both tensile strength and elongation at break increased markedly. This enhancement implies that improved RM dispersion and interfacial compatibility at higher contents promoted more effective stress distribution and cooperative deformation between PBAT and PLA phases, resulting in simultaneous strengthening and toughening in TD, similar to Joncryl in PLA/PBAT blends [32].

### 2.4. Morphological Analysis

**Figure 6:**
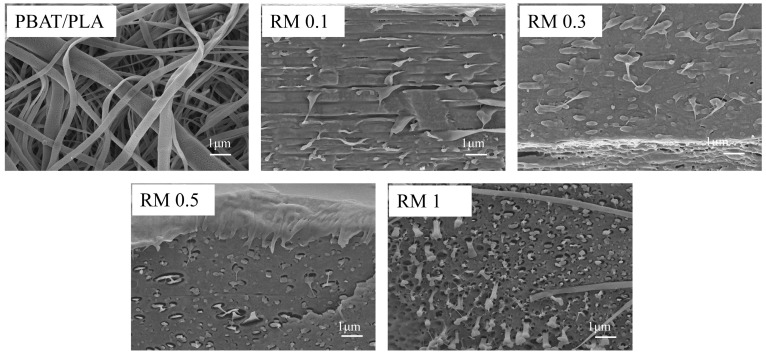
SEM micrographs of PBAT/PLA/RM films.

SEM was employed to characterize the microstructure of PBAT/PLA films (Figure 6) with varying RM concentrations, and distinct morphological evolutions were observed. The neat PBAT/PLA blend exhibited a typical immiscible structure, with discrete domains of the dispersed phase (predominantly PBAT) randomly distributed in the continuous PLA matrix. These domains featured relatively large sizes and distinct phase boundaries, indicating poor interfacial adhesion between the two polymers.

The fracture behavior of PBAT/PLA blends transitioned from ductile fracture to fiber debonding or pore formation with variations in RM concentration. The neat PBAT/PLA blend exhibited ductile fracture, characterized by obvious plastic deformation and continuous matrix tearing during tensile testing. At low RM concentrations (0.1–0.3%), the blend retained a certain degree of ductility, with the fracture surface showing cohesive failure of the matrix. As RM concentration increased to 0.5–1%, the enhanced interfacial compatibility modified the stress transfer mechanism: the dispersed phase particles (PBAT) tended to debond from the PLA matrix under external force, and the separation of these particles led to the formation of tiny pores on the fracture surface. Eventually, the fracture mode shifted from uniform ductile deformation to localized fiber debonding accompanied by discrete pore generation, which was closely related to the interfacial interaction and microstructure evolution regulated by RM.

### 2.5. UV-Vis Transmittance

**Figure 7:**
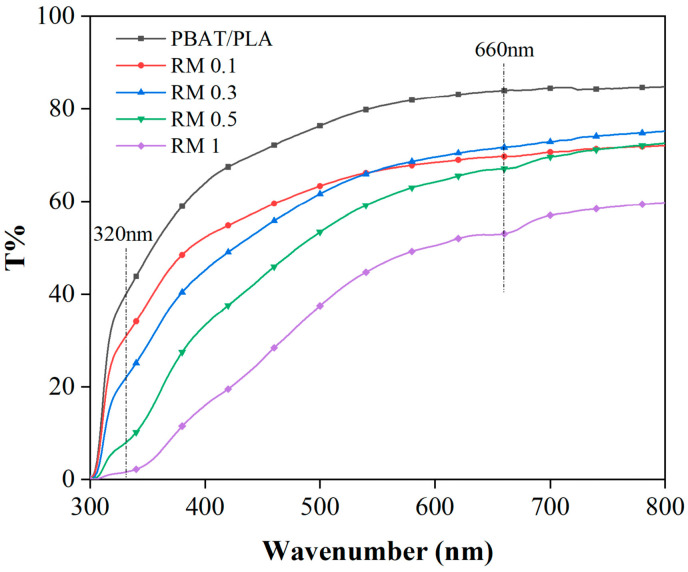
UV-Vis transmittance of PBAT/PLA/RM blend films.

The UV–visible region transmittance of the PBAT/PLA/RM composite films is shown in Figure 7. It can be observed that the transmittance of PBAT/PLA films in the visible region is more than 80% (in terms of UV transmittance at 660 nm), and the transmittance in the UV region is also higher, which indicates that the UV absorption performance of PBAT/PLA/RM composite films is poor. The transmittance of the films in the visible region was reduced to 60% with the addition of 0.1–0.5% rosemary extract compared with that of PBAT/PLA, which indicates that the addition of rosemary extract from 0.1% to 0.5% has less effect on the transmittance of PBAT/PLA films in the visible region. The typical visible light transmittance of the PBAT/PLA/RM composite film is 50% at 1%, which is a significant decrease in the transparent visibility compared to PBAT/PLA, and therefore more suitable for application scenarios with lower transparency requirements. With the increase in rosemary extract, the UV-blocking ability of PBAT/PLA/RM is incrementally enhanced, and more than 90% of UVA and UVB can be filtered when the content of rosemary extract is more than 0.5%. In summary, the addition of rosemary extract provides the film with excellent UV-blocking performance.

### 2.6. Antioxidant Property

**Figure 8:**
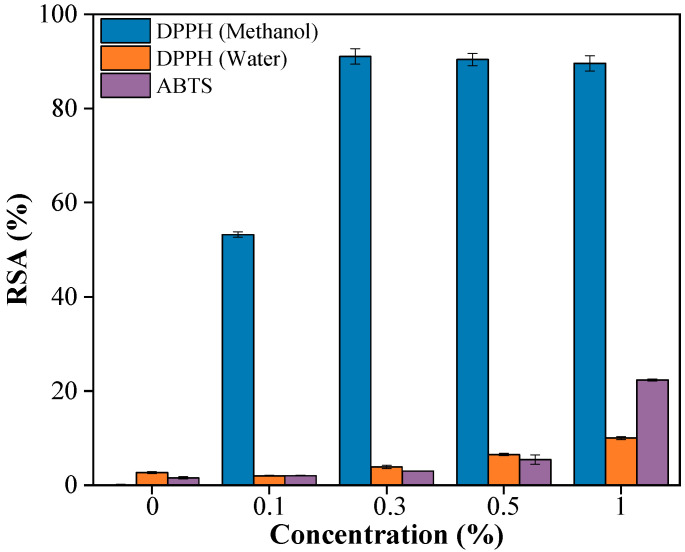
Antioxidant property of PBAT/PLA/RM films with different RM concentrations (0, 0.1, 0.3, 0.5, 1).

The antioxidant activity of PBAT/PLA/RM films was evaluated using DPPH (methanol and water as extraction solvent respectively) and ABTS assays (Figure 8), revealing that the antioxidant activity strongly depends on both RM content and the polarity of the extraction solvent. In the DPPH–methanol system, RSA increased rapidly with RM content and exceeded 90% at 0.3% RM, indicating efficient extraction of antioxidant components from RM, whereas the pristine PBAT/PLA film showed negligible activity. In contrast, when water was used as the extraction medium, the scavenging efficiency remained low, reaching only ~10% at 1% RM, which can be attributed to the limited solubility and migration of predominantly hydrophobic antioxidant compounds in RM. The ABTS assay showed a gradual increase in radical scavenging activity with increasing RM content, reaching approximately 22% at 1% RM, reflecting its ability to respond to both hydrophilic and lipophilic antioxidants. Overall, these results confirm that PBAT/PLA/RM films possess antioxidant functionality, while the observed polarity-dependent activity suggests that the release of RM-derived antioxidants can be modulated by the surrounding environment, which is relevant for potential food-packaging applications.

### 2.7. Antibacterial Property

**Figure 9:**
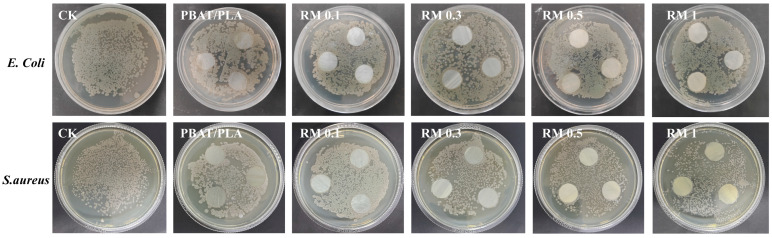
Antibacterial performance of PBAT/PLA/RM films.

The antibacterial activity of PBAT/PLA films with different RM concentrations was assessed against *E. coli* and *S. aureus* with the control (CK) and neat PBAT/PLA film as references (Figure 9). The results showed that the neat PBAT/PLA films had negligible antibacterial effect, while RM-modified blends exhibited varying degrees of antibacterial activity against both strains. For *E. coli*, the antibacterial efficiency increased with RM concentration: RM 0.1% showed slight inhibition, while 1% exhibited more pronounced antibacterial effects. A similar trend was observed for S. aureus, with RM 1% showing the highest antibacterial activity.

The antibacterial effects of RM-modified films may be attributed to several factors: (1) functional groups in RM (e.g., epoxy or cationic groups) could interact with bacterial cell membranes, potentially compromising their integrity; (2) RM may release active components that inhibit bacterial metabolism or DNA replication; and (3) the improved interfacial structure of the blends may hinder bacterial adhesion and proliferation [35]. The observed concentration-dependent antibacterial activity generally paralleled the antioxidant performance, suggesting that RM content may influence both properties [36]. Although the specific link between RM concentration, active sites, and antibacterial or antioxidant effects was not directly measured in this study, higher RM content appeared to enhance both activities. This enhancement of antibacterial activity in RM-modified PBAT/PLA blends may be beneficial for biodegradable packaging applications, potentially reducing the risk of bacterial contamination and extending the shelf life of food products.

### 2.8. Soil Burial Compostability

**Figure 10:**
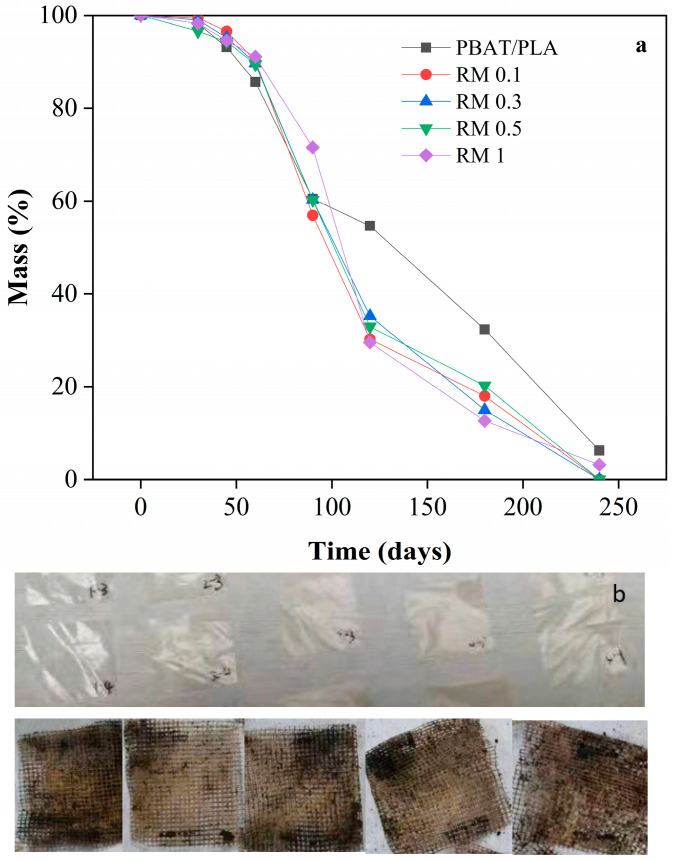
Compost degradability of PBAT/PLA/RM composite films: pictures of PBAT/PLA/RM composite films before (**a**) and after compost (**b**).

The results of compost degradability of PBAT/PLA/rosemary extract blend film over 240 days are shown in Figure 10. As observed from the figure, the first 60 days of composting represent a critical stage of fermentation and microorganism enrichment in the composting medium. During this period, the degradation of all samples was very slow, with a mass loss rate of less than 5%. This indicates that the initial microbial activity and breakdown were minimal.

After 90 days, all samples showed approximately 40% degradation. The composting medium maintained a water content of 35%, facilitating microbial activity. The microorganisms adhering to the surface of the PBAT secreted hydrolases, which bound to the PBAT surface, breaking the macromolecular chains into smaller molecules. This enzymatic activity significantly increased the weight loss rate. Comparing the mass loss rates, the PBAT/PLA in the blank group exhibited a lower degradation rate than the rosemary extract-added group after 90 days. This indicates that the rosemary extract promoted the degradation of the film. The small molecular compounds resulting from the degradation process were ingested by microorganisms and ultimately converted into CO_2_ and H_2_O.

After 240 days, the samples with the addition of rosemary extract were fully degraded, whereas approximately 5% of the blank group remained. This suggests that the rosemary extract significantly enhanced the degradability of the blend film. The probable reason for the low degradation rate of all samples within 90 days was that the addition of rosemary extract inhibited the growth activity of microorganisms, thus resulting in a slow degradation rate of all samples. However, after 240 days of composting, all samples met the criteria for degradability as defined in the standard for biodegradation of more than 90%.

## 3. Experimental

### 3.1. Materials

PBAT (TH801T) of density 1.27 g/cm^3^ was purchased from Xinjiang Lanshan Tunhe Chemical Co., Ltd., Changji, China; PLA (FY801) of density 1.25 g/cm^3^ was provided by Anhui BBCA Biochemical Futerro PLA Co., Ltd., Bengbu, China; Polycaprolactone (PCL) was provided by Perstorp, Sweden; 2,2-Biphenyl-1-picrylhydrazyl (DPPH) of 97% purity, Methanol (CH_3_OH) of purity > 99.9% and Dichloromethane (CH_2_Cl_2_) of purity > 99.7%, 2,2′-azino-bis(3-ethylbenzothiazoline-6-sulfonic acid) (ABTS) of purity > 98% and potassium persulfate (K_2_S_2_O_8_) of purity > 99% were provided by Aladdin Reagent (Shanghai) Co., Ltd., Shanghai, China. *Rosmarinus officinalis* L. (Rosemary Extract, Food grade, fat-soluble, mainly containing rosmarinus acid and carnosic acid, of which the content of sage acid is about 10–15%) was provided by Henan Senbenben Grass Natural Products Co., Ltd., Zhengzhou, China; *Escherichia coli* (*E. coli*, DH5α) and *Staphylococcus aureus* (*S. aureus*, RN4220), were purchased from Changsha Abiowell Biotechnology Co., Ltd., Changsha, China and ThermoFisher Scientific, Waltham, MA, USA, respectively.

### 3.2. Preparation of PLA/PBAT/RM Masterbatch

The natural antioxidant–rosemary extract (RM) was dried in the airdrying oven at 60 °C for 24 h, respectively, and PBAT, PLA and rosemary extract were mixed by a mixer in a certain proportion to prepare the masterbatch containing rosemary extract and the blank group of masterbatch, and PBAT/PLA/rosemary extract and PCL were mixed uniformly and put into a twin-screw extruder (KY mach SJ-30, Nanjing, China) for extruding and pelletizing, in which the temperature of twin-screw extruder was 155, 165, 170 and 160 °C from zone 1~4, respectively; the naming and composition of masterbatch are shown in Table 3. Here PCL served as a compatibilizer to improve the compatibility of the polymer matrix, ensuring a more uniform distribution during melt blending and extrusion.

### 3.3. Preparation of PBAT/PLA/RM Extract Degradable Composite Films

PBAT, PLA and masterbatch were mixed in specific proportions to maintain a PBAT/PLA ratio of 70:30 in the blank sample. For the experimental groups, the mass fractions of RM were 0.1%, 0.3%, 0.5% and 1%, respectively. The pre-prepared masterbatch and the raw PBAT and PLA were mixed and extruded to be the film by the film blowing machine with a screw diameter 170 mm and an L/D ratio of 30 (Leibo, Hangzhou, China). The film-blown temperature was independently controlled at four zones along the extruder barrel, and a strand die was used to achieve a temperature profile in the range of 150–160 °C, finally five types of films were obtained (thickness = 25–35 μm): the blank control, the films with the mass percentage of RM of 0.1, 0.3, 0.5, and 1, respectively. One important observation shall be reported here: the reason we stopped at 1% of RM is because it is found that higher concentration of RM will make blends very sticky and difficult to be processed for film blowing (we have performed the experiments with 1.2 and 1.5%). This drawback may limit some of the expected properties of the film, which will be discussed later.

### 3.4. Characterization

#### 3.4.1. Characterization of Rosemary Extract

Fourier transform infrared (FTIR) spectra of the rosemary extract were recorded using an FTIR spectrometer (ALPHA II, Bruker, Bremen, Germany) in attenuated total reflection (ATR) mode. The measurements were performed over the wavenumber range of 4000–600 cm^−1^ with a resolution of 4 cm^−1^. For each sample, the sample was placed directly on the diamond crystal of the ATR accessory, and 32 scans were accumulated to obtain the spectrum.

The thermal stability of rosemary extract was evaluated using a thermogravimetric analyzer (STA200, HITACHI, Tokyo, Japan). Approximately 10 mg of the sample was placed in a ceramic crucible and heated from 25 °C to 600 °C at a constant heating rate of 10 °C/min under a nitrogen atmosphere. The derivative thermogravimetric (DTG) curve was obtained by differentiating the TGA data. The initial decomposition temperature (T_onset_) and maximum decomposition temperature (T_m_) were determined from the DTG curve, while the residual char content (%) at 600 °C were calculated from the TGA curve.

An Agilent (Santa Clara, CA, USA) Technologies 8890A Gas Chromatograph (GC) equipped with a 5977C mass selective detector (MSD) was used for analysis in electron impact (EI) mode at 70 eV. Helium was employed as the carrier gas, and the sample was separated using an HP-5 capillary column (30 m × 0.25 mm, 0.25 μm). Data analysis was performed using GC/MSD Chemstation software B.04.01 and MestreNova 14.0.

The GC oven temperature program began at 50 °C for 2 min, then increased at a rate of 2 °C/min to 80 °C for 1 min, followed by a ramp of 8 °C/min to 100 °C for 1 min, and a further ramp of 10 °C/min to 200 °C for 1 min. Finally, the temperature was raised to 300 °C at 10 °C/min and held for 5 min. The carrier gas flow rate was set at 1.0 mL/min in splitless mode, with the mass spectrometer operating in the *m*/*z* range of 40–1000.

The sample was diluted 1:40 in ethyl acetate, and 1 μL was injected into the GC. n-Octane (99.8% purity) was used as an internal standard at a final concentration of 0.3 mg/mL. Alkanes (C8–C24) were analyzed under the same conditions to calculate retention indices using the Van den Dool and Kratz equation. The results were expressed as the percentage of the peak area for each compound relative to the internal standard, with identification confirmed using the NIST database.

#### 3.4.2. Thermal Property

The thermal properties of the films were tested using a differential scanning calorimeter (DSC 200, Hitachi, Japan). Film samples (8–10 mg) were sealed in aluminium pans with lids and heated from −50 to 200 °C at a heating rate of 10 °C/min under a nitrogen atmosphere with a flow rate of 50 mL/min. The glass-transition temperature (T_g_), crystallization temperature (T_c_), and melting temperature (T_m_) of film samples were determined from the thermograms. T_m_ and T_c_ were taken as the peak values of the respective endotherms, and T_g_ was taken as the midpoint of the heat capacity changes. A three-cycle thermal program (heating, cooling, reheating) was performed to eliminate thermal history.

A thermogravimetric analyzer was used to track the thermal decomposition process of the film with increasing temperature in the presence of protective gas, and the thermal stability of the composites was analyzed by the heat loss curve obtained. Test conditions: the heating rate was 10 °C/min, the sample weight was 8~10 mg, the test was carried out under nitrogen atmosphere, and the nitrogen protective purge rate was 20 mL/min. In addition, the composites were dried in an oven at 60 °C to remove the moisture before the test to avoid the influence of moisture on the experimental results.

#### 3.4.3. Tensile Test

Tensile strength and elongation at break in the machine direction (MD) and transverse direction (TD) of the produced films were investigated by using a universal testing machine (GBH-1, Biaoji, China), according to the GB/T 1040.3-2006 standard [37]. The pre-conditioned films were cut into 150 mm × 15 mm (L × W) strips and the measurements were carried out at a crosshead speed of 300 mm/min, a load cell of 500 N and an initial gauge length of 50 mm. At least six replicates were tested for each formulation and the average values were reported.

#### 3.4.4. Morphology

Blend films were fractured in liquid nitrogen to observe the interior of the unstressed composite. A sample was dropped directly into liquid nitrogen and fractured with a pre-chilled razor blade held in a vice-grip. The fractured pieces were picked out of the liquid nitrogen using pre-chilled forceps and placed in a desiccator to thaw and reduce the condensation of water on the surface of the material. All specimens were coated with gold-palladium for 45 s. Specimens were viewed in a scanning electron microscope (Zeiss Sigma 360, Oberkochen, Germany) at 3 kV.

#### 3.4.5. UV–Visible (UV-Vis) Transmittance

The light transmittance of the film was tested by UV 5200PC (Shimadzu, Tokyo, Japan) UV–visible spectrophotometer with a scanning step rate of 240 nm/min and a wave number scanning range of 300–800 nm.

#### 3.4.6. Antioxidant Activity

The antioxidant action of PBAT/PLA/AAE films were determined using three different assays, DPPH (methanol and water were used as immersing solvents respectively) and ABTS radical scavenging methods.

##### DPPH

The absorbance of the aqueous solution was measured using a UV-Vis spectrophotometer (UV 5200PC, Shimadzu, Japan) over a wavelength range of 400–800 nm, the characteristic absorbance and the reduction in the DPPH radical signal at 515 nm were recorded and calculated according to a previously established method [38], with the detailed experimental procedure outlined below. Films were fragmented and immersed in methanol at a ratio of 1 g/20 mL. The mixture was consistently agitated until the films thoroughly immersed into methanol and then allowed to stand at room temperature for 24 h. The supernatant was collected and mixed with the methanolic solution of DPPH (50 mg/L). Finally, the resultant mixture was agitated thoroughly and maintained in darkness for 1 h, after that the absorbance of the solution was monitored at 515 nm. Methanol was used as the blank, while DPPH and methanol was set as negative control. The ability to scavenge DPPH radical was expressed as the percentage of reduction of the free radical by the sample and calculated as:

(1) $$\mathrm{Radical}\;\mathrm{scavenging}\;\mathrm{activity}\;(\mathrm{RSA},\;\%)\;=\;\frac{A_{control\;-{\;A}_{sample}}}{A_{control}}\times 100$$

where A_control_ is the absorbance of 25 mg/mL DPPH/methanol solution at 515 nm. A_sample_ is the absorbance of specified sample at 515 nm.

In the case of water as immersing solvent, every sample (25 mg) of each film was immersed in 3 mL of distilled water, and then 2.8 mL of film extract solution was mixed with 0.2 mL of 0.3 mM methanolic solutions of DPPH. The absorbance at 515 nm was measured after the solution had been allowed to stand in the dark at ambient temperature for 30 min [39]. The percentage of DPPH radical-scavenging activity was calculated using Equation (1).

##### ABTS Assay

A 7 mM ABTS solution was mixed with 2.45 mM K2S2O8 in 1:1 ratio for 16 h at 25 °C in the dark. The solution was then diluted with ethanol until the absorbance of about 0.70 at 734 nm was achieved, which was then denoted as A0. About 50 mg of the film samples were added to 10 mL of the ABTS solution and incubated at room temperature in the dark for 30 min [13,40]. After incubation, the absorbance at 734 nm was measured (AT) and the antioxidative activity of the film sample was calculated as follows:

(2) $$\mathrm{Free}\;\mathrm{radical}\;\mathrm{scavenging}\;\mathrm{activity}\;(\%)\;=\;\frac{A_{0-}A_{T}}{A_{0}}\times 100$$

where A_0_ and A_T_ were the absorbance of ABTS of the control and test film, respectively.

#### 3.4.7. Antibacterial Test

Gram-positive (*S. aureus*) and -negative bacteria (*E. coli*) were used as experimental strains to evaluate the antibacterial effect of blend films. The bacterial strains were first cultured to the logarithmic growth phase, then diluted to a final concentration of 1 × 10^4^ CFU/mL. The concentration of bacterial suspensions was verified by measuring their absorbance at 600 nm using a microplate reader. For colony growth observation, 100 μL of the standardized bacterial suspension was evenly spread on Luria–Bertani (LB) agar plates. Each group of blend films was then placed on the agar surface, and the Petri dishes were incubated at 37 °C for 48 h to assess the antibacterial effect.

#### 3.4.8. Soil Burial Test of Films

The disintegrability of PBAT/PLA/RM films in simulated composting conditions was tested according to the European standard ISO 20200:2015 [41]. A specific quantity of compost was mixed with the synthetic biowaste prepared with sawdust, rabbit food, starch, sugar, oil and urea. The water content of the substrate was around 35 wt% and the aerobic conditions were guaranteed by mixing it gently. The samples (30 mm × 30 mm × 20 μm) were buried at 4–6 cm depth in perforated boxes containing the prepared mix and incubated at 58 °C during 17 days. The samples were recovered at different intervals, washed with distilled water, dried in an oven at 35 ± 2 °C for 3 days, and weighed. The degradation values (wt%) at different periods of incubation were obtained by normalizing the sample weight to the initial weight.

## 4. Conclusions

In this work, we developed and characterized rosemary extract-infused PBAT/PLA biodegradable composite films using melt extrusion and film blowing techniques. The films demonstrated enhanced antioxidant, UV-blocking, and thermal stability properties, while maintaining industrial relevance in terms of processability and compostable end-of-life behavior.

First, the processing feasibility of PBAT/PLA/RM films was determined: RM concentrations above 1% led to excessive stickiness of the blown films, making smooth processing impossible. Thus, 1% was identified as the maximum viable RM loading for subsequent experiments. Second, RM significantly improved the antioxidant activity of the composite films, with the optimal performance observed at 0.3% RM, where the films exhibited strong DPPH free radical scavenging capacity. Third, the UV-shielding property of PBAT/PLA films was effectively enhanced by RM, with higher RM concentrations resulting in better UV-blocking effects—films containing ≥0.5% RM achieved over 90% filtration of UVA and UVB, expanding their applicability to light-sensitive food packaging. Fourth, contrary to expectations, the antimicrobial efficacy of RM was limited; notably, the 1% RM sample showed an increased number of *Escherichia coli* colonies, which may be attributed to suboptimal RM concentration or inactivation of bioactive compounds during thermal processing. Fifth, regarding biodegradability, RM initially inhibited microbial activity in the composting medium, prolonging the overall degradation period of the films. Nevertheless, all composite films with varying RM contents achieved over 90% degradation after 240 days, complying with biodegradability requirements.

Overall, this research highlights the potential of RM as a natural additive to enhance the functional properties of PBAT/PLA films, particularly in terms of antioxidant activity and UV resistance. However, challenges remain, including the need to improve antimicrobial performance and optimize processing conditions to mitigate film stickiness at higher RM loadings. Future studies should focus on adjusting RM concentrations, exploring combinations with other natural antimicrobial agents, optimizing processing parameters, and evaluating the practical application of these films in preserving diverse food products. These efforts will contribute to the development of high-performance, eco-friendly food packaging solutions that reduce environmental plastic waste while ensuring food safety and quality.

## Figures and Tables

**Table 1:** List of volatile compounds in rosemary extract. Their % content is presented along with the experimental and literature retention indices.

No.	Volatile Compounds	% Peak Area/IS Area
**1**	3-Pentanone	3.59
**2**	Butane, 1-ethoxy	4.14
**3**	Octane	24.59
**4**	Bicyclo[2.2.1]heptan-2-ol, 1,7,7-trimethyl-, (1S-endo	0.67
**5**	Bicyclo[3.1.1]hept-3-en-2-one, 4,6,6-trimethyl	3.08
**6**	14-Hydroxycaryophyllene	0.54
**7**	Silane, dimethyl(4-(2-phenylprop-2-yl)phenoxy)butoxy	1.39
**8**	Ferruginol	2.05
**9**	Phenol, 2,6-bis(1,1-dimethylethyl)-4-[(4-hydroxy-3,5-dimethylphenyl)methyl]-	7.72
**10**	Isocarnosol	22.59
**11**	4-Cumylphenol, TMS derivative	21.65
**12**	12-O-Methylcarnosol	1.44
**13**	4H-1-Benzopyran-4-one, 5-hydroxy-7-methoxy-2-(4-methoxyphenyl)	3.19
**14**	dl-.alpha.-Tocopherol	1.53
**15**	4H-1-Benzopyran-4-one, 5-hydroxy-6,7-dimethoxy-2-(4-methoxyphenyl)-	1.81

**Table 2:** Characteristic decomposition temperature (T_onset_ and T_m_) of films.

RM Concentration (%)	T_onset_	T_m1_	T_m2_	T_m3_	T_m4_
PBAT/PLA	323.5	349.5	388.5	466.5	479.5
0.1	317	349.5	388.5	453.5	473
0.3	323.5	349.5	388.5	466.5	-
0.5	323.5	349.5	382	479.5	-
1	323.5	343	388.5	479.5	-

**Table 3:** Formulation of PBAT/PLA/RM masterbatch.

	PBAT (%)	PLA (%)	PCL (%)	RM (%)
PBAT/PLA/RM	25	70	2	3
Control	28	70	2	/

## Data Availability

The main data generated or analyzed to support the conclusion during this study are included in this published article. The full datasets generated and/or analyzed during the current study are available from the corresponding author by request.
